# Valorization of Sweet Lime Peel for the Extraction of Essential Oil by Solvent Free Microwave Extraction Enhanced with Ultrasound Pretreatment

**DOI:** 10.3390/molecules25184072

**Published:** 2020-09-07

**Authors:** Yasir Arafat, Ammar Altemimi, Salam Adnan Ibrahim, Laxmikant Shivnath Badwaik

**Affiliations:** 1 Department of Food Engineering and Technology, School of Engineering, Tezpur University, Napaam 784028, Assam, India; yasir.arafatt@gmail.com; 2 Department of Food Science, College of Agriculture, University of Basrah, Basrah 61004, Iraq; ammaragr@siu.edu; 3 Food and Nutritional Science Program, North Carolina A & T State University, Greensboro, NC 27411, USA; ibrah001@ncat.edu

**Keywords:** microwave extraction, essential oil, ultrasound treatment, sweet lime peel, antimicrobial activity

## Abstract

Essential oils of sweet lime peel, a waste by-product in the juice industry, were extracted using the vacuum assisted solvent free microwave extraction (VASFME) method. The effects of microwave output power (500–1000 W) and extraction time (20–30 min) on the essential oils yield and antimicrobial property were investigated. Optimal conditions were observed at 797.844 W microwave output power and 30 min extraction time. The essential oils yield and antimicrobial property under these conditions were 0.792 ± 0.03% and 18.25 ± 1.45 mm, respectively, which agrees with the predicted values of 0.757% and 16.50 mm. The essential oils were extracted at optimized conditions and analyzed through GCMS for compound identification. A total of 49 compounds were identified, with limonene content (43.47%) being the highest among all sweet lime peel oil compounds. Moreover, the sweet lime peels were subjected to ultrasound pre-treatment before microwave extraction. The ultrasound pre-treatment helped to increase the essential oils yield from 0.84 to 1.06% as the treatment time increased from 30 to 90 min. The increase in yield was 37.66% more compared to VASFME at 90 min treatment time.

## 1. Introduction

The sweet lime (*Citrus limetta*) is a native plant of Asia. It is a rich source of Vitamin C and energy, and it is consumed fresh or squeezed to make juice. Around the world, citrus juices are the most common and popular fruit juices among consumers [1]. The peel of citrus fruit is considered as a by-product for some useful application. However, sometimes, the peel is treated as a waste material. In this case, it can cause water pollution due to the presence of various biomaterials [2]. Sweet lime peel is a source of essential oils, flavonoids and pectin [3]. The oil extracted from citrus peel has a strong and desirable aroma with a refreshing effect and has been used as a flavoring in foods, beverages and pharmaceutical products. Apart from this, citrus peel essential oils have been recognized as safe due to their wide spectrum of biological activities such as antimicrobial, antioxidant anti-inflammatory and anxiolytic [4].

Essential oils are the volatile fraction of the secondary metabolites produced by plants and have a specific narcotic odor, are colorless, highly refractive and easily inflammable. Such volatile constituents are mainly a mixture of terpenes and terpenoids. Monoterpenes are the most representative essential oils molecules constituting 90% of the total. However, as essential oils are highly complex, they may also include oxygenated compounds such as alcohols, aldehydes, ketones, acids, phenols, oxides, lactones, acetals, ethers and esters [5].

Nowadays, consumer food preferences tend more toward natural, less processed products and chemical-free additive formulation. Many naturally occurring compounds found in edible and medicinal plants, herbs, and spices have been shown to possess antimicrobial functions and are thus useful as antimicrobial agents. Essential oils from such plant sources have shown antimicrobial activities against spoilage microorganisms, foodborne and postharvest pathogens [6].

The majority of essential oils come from plant sources in which different methods of distillation or cold pressing are employed to extract peel oils from the citrus fruit. Steam distillation is the primary method used to extract essential oils from plant sources [7]. In addition, several other conventional methods such as soxhlet extraction, hydro-distillation and simultaneous distillation extraction are used to extract the essential oil [8,9]. Distillation methods used very high temperature for extraction of desirable compounds. Essential oils molecules are very sensitive to heat due to its low boiling point and are thus vulnerable to chemical changes. As a result the extraction methods can lead to the loss of some volatile compounds and degradation of unsaturated or ester compounds through thermal effects [10]. Whereas, the use of petroleum-based solvents for essential oil extraction is characterized with high energy and time consumption, lower product yield, and generation of hazardous by products [11]. These shortcomings have led to the consideration of the use of new techniques in essential oil extraction, which typically useless solvent or no solvent and less energy. New techniques such as supercritical fluid extraction, ultrasonic and microwave extraction have been found to have high potential of overcoming the drawbacks associated with other conventional methods [12].

Microwave extraction is well known for selective and volumetric heating of the target, and it uses less solvent or no solvent, with less extraction time and a higher product quality. In the microwave extraction process, microwave heating and distillation are combined as materials (e.g., plant materials) are subjected to extraction without the use of a solvent. The glands and oleiferous receptacles of plant materials burst due to heating of in situ water which helps to free the essential oil along with production of water vapor. The water vapor and essential oil condense and are collected in the receiving flask. The water and essential oil layer can be easily separated and collected without the addition of any solvents [13]. Many researchers utilized citrus industry waste for extraction of essential oil by microwave extraction process and other methods [12,14,15,16,17,18].

The food processes resulting from exposure to ultrasound are believed to be affected in part by cavitation phenomena and mass transfer enhancement. The ultrasonic waves can cause a rapid series of alternative compressions and expansions in materials and result in the creation of microscopic channels. During continuous rarefaction and compression cycles, cavitation bubbles are produced and an increase in temperature and pressure occurs. This leads to a collapse of the bubble. A shock wave occurs in the solvent which causes greater penetration of the solvent into the plant materials, and more extraction takes place [19].

In the current study, extraction of sweet lime peel essential oil was done by vacuum assisted solvent free microwave extraction (VASFME) method. The effects of process parameters such as extraction time and microwave power input were investigated on essential oil yield and antimicrobial property. In addition, the effect of ultrasound pre-treatment followed by microwave assisted extraction on the yield of essential oil was investigated.

## 2. Results and Discussion

### 2.1. Extraction of Essential Oil from Sweet Lime Peels

Moisture content of the raw sweet lime peels and crude fat content of the dried peels were observed as 72.4 ± 0.12% and 1.6 ± 0.06%, respectively. The raw sweet lime peels were taken for the vacuum assisted microwave extraction (VASFME) process. Response surface methodology was used to determine the effect of microwave power (500–1000 W) (X_1_) and time of exposure (20–30 min) (X_2_) on essential oils yield and antimicrobial property. The range of microwave power and extraction time was selected based on different experimental trials. The results are shown in Table 1.

### 2.2. Model Fitting

The coefficient of regression of the intercept and linear terms of the model were calculated using the least square technique. Regression analysis and ANOVA were used for fitting the model and for examining the statistical significance of terms. The results of ANOVA are given in Table 2. The correlation coefficients (R^2^) for the essential oil yield and antimicrobial property were 0.9498 and 0.8903, respectively. Based on t-statistics, the regression coefficient significant at 95% probability levels was selected for developing the model below (Equations (1) and (2)). These equations were developed to show the relationship between independent variables (microwave power and extraction time) on dependent variable (yield and antimicrobial activity of essential oils) [12,20].

(1) $$\mathrm{Yield}=0.70+0.064X_{1}+0.045X_{2}$$

(2) $$\mathrm{Antimicrobial}\;\mathrm{property}=20.54-13.33X_{1}-1.50X_{2}$$

where, X_1_ and X_2_ are microwave power (W), extraction time (min) and each variable is set in the range of 500–1000 W, 20–30 min, respectively. The lack of fit was not significant (*p* > 0.05), and the model F values were 94.64 and 40.59. There is only a 0.01% chance that a model F value could occur due to noise. The coefficient of variation (C.V.) was 1.99% and 17.76%, suggesting that the model was reliable and reproducible. The results thus indicated that the model could work well for the prediction of essential oil yield and antimicrobial property from sweet lime peels.

### 2.3. Effect of Process Variables on Responses

**Figure 1:**
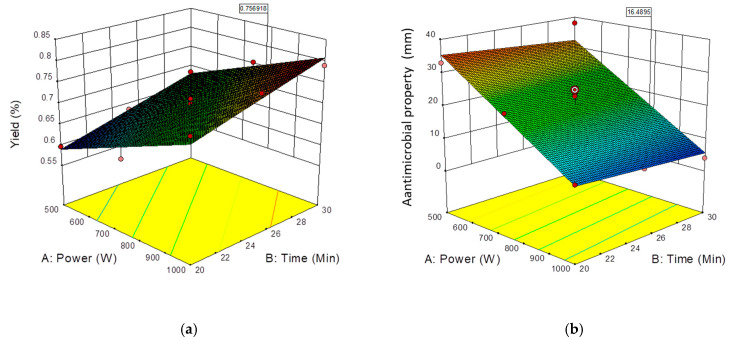
Effect of process variable on (**a**) yield (5) and (**b**) antimicrobial property (mm) against *E. coli* of essential oil.

The effects of microwave power and extraction time on essential oil yield using fresh sweet lime peel are shown in Figure 1a. Fresh samples of 400 g each were extracted with microwave power levels at 500–1000 W for 20–30 min. Both microwave power and extraction time had a significant effect on essential oil yield (*p* ≤ 0.01) (Table 2). The yield was obtained in the rage of 0.60 to 0.79 % on fresh peel weight basis. The current extraction efficiency was found high compared to the essential oils yield reported for orange peel (0.40 % dry basis in 30 min) using solvent-free microwave assisted extraction [21]. It has been observed that both microwave and extraction time have a positive effect on yield although the effect of microwave power is the greater of the two. The observed effect is same with results reported Auta, Musa [12] for citrus peels essential oil extraction. They had reported yield of essential oil of 3.7% for orange and 2.5% lemon on dry weight basis. Increased power levels resulted in higher oil yield, but the quality of the oils degraded [22]. This fact can be attributed to overheating, which leads to the degradation of heat-sensitive compounds [23].

Microwave power has a significant effect on the antimicrobial property of essential oil at *p* ≤ 0.01, but extraction time is not significantly affected (Table 2). Microwave power and extraction time both have a negative effect on the antimicrobial property of essential oils, but the effect of extraction time was not substantial. Antimicrobial property decreases with increasing microwave power. This may be due to the destruction of active compounds at high power levels [24]. Figure 1b shows the effects of microwave power and extraction time on the antimicrobial property of essential oils.

### 2.4. Optimization of Extraction Process

The numerical optimization technique was used for optimization of extraction process based on the desirability approach. This technique was carried out in order to obtain the maximum essential oil yield and antimicrobial property and thus helped to determine the optimum condition for the extraction of essential oil from sweet lime peel. For optimization, microwave power and extraction time was kept in range and maximized the essential oil yield and antimicrobial property. The optimized microwave power value for the extraction of essential oil was found to be 797.844 W for 30 min. The predicted experiment values for the essential oil yield and antimicrobial property were 0.757% and 16.50 mm, respectively, and the actual experiment values were 0.792 ± 0.03% and 18.25 ± 1.45 mm, respectively.

### 2.5. Effect of Ultrasound Pre-Treatment on Essential Oil Yield

Ultrasound pre-treatment had a significant impact on the yield of essential oils from sweet lime peel. The yield varied from 0.84 to 1.06% with ultrasound pre-treatment followed by vacuum assisted solvent free microwave extraction (VASFME) and was significantly higher than that of the only vacuum assisted solvent free microwave extraction at (*p* ≤ 0.05). This may be due to breakdown of plant matrix and the release of intracellular components due to cavitation phenomenon that can cause the high shear force in samples due to ultrasound pre-treatment process [25]. Table 3 shows variation of essential oil yield with respect to treatment time. The achieved yields were higher in ultrasound traded VASFMW than that of VASFMW. However, the increase in yield was 7.69%, 16.67%, 37.66% and 23.38% for the peels exposed to ultrasound for 30, 60, 90 and 120 min, respectively. Ultrasound pre-treatment before extraction is capable of enhancing the extraction efficiency and shortening the processing time [26]. In all experiments, the extraction yield was (*p* ≤ 0.05) time dependent. The yield increased with extended ultrasonic times but slowly decreased from 90 to 120 min due to the squeezing out of essential oil from the solvent media as a result of the prolonged ultrasound treatment. The efficient extraction period for achieving the maximum yield was 90 min which was 37.66 % more compared to VASFME. This difference can be attributed to the fact that extraction requires two stages. The first stage involves the penetration of the solvent into the cellular structure followed by the dissolution of soluble constituents in the solvent. The second stage involves the external diffusion of soluble constituents through the porous structure of the residual solids and its transfer from the solution in contact with the particles to the bulk of the solution [27]. The use of ultrasonic pre-treatment helps to reduce the extraction time and enabled a growth in the extraction of bioactive compounds and consequently improving the antioxidant and antimicrobial activities of essential oils [28].

### 2.6. Physical Properties and Composition of Essential Oil

The specific gravities and refractive indices of sweet lime peel essential oil extracted by VASFME under optimum conditions were 0.864 ± 0.002 and 1.463 ± 0.001, respectively. These physical properties are comparable with findings reported by Chenni, El Abed [29] for sweet basil essential oil obtained by solvent free microwave extraction process. The composition of essential oil from sweet lime peel obtained at the optimum condition was analyzed by GC-MS. A total of 49 compounds were identified by mass spectrometry. Out of that the seven major compounds of sweet lime peel essential oil are shown in Table 4. The compound d-limonene showed a highest concentration (43.47%), followed by bergamol (7.26%), β-pinene (6.84%), linalool (5.60%), α-pinene (4.49%), 1,8 cineole (4.05), α-terpineol (3.16) and the remaining compounds are in very minor quantity. The composition of essential oil compounds are in line with study reported by Megha and Mumtaj [1] for sweet lime peel using microwave-assisted hydrodistillation. However, proportion of d-limonene reported by them is quit higher (78.3%). The higher microwave extraction power used in this study might affect the yield of d-limonene. The compounds found in sweet lime peel oil are also in agreement with previous reported composition [3,30].

## 3. Materials and Methods

### 3.1. Raw Materials

Sweet lime peel was collected from a juice vendor at Tezpur University, Assam, India and stored in a refrigerator. The peels were cut into pieces (about 1 cm length) with a knife prior to extraction and analyzed for moisture content and crude fat content [31].

### 3.2. Microwave Extraction Setup

**Figure 2:**
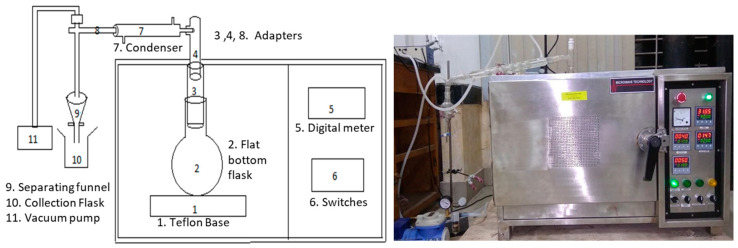
Schematic diagram and actual picture of microwave extraction set up.

Vacuum-assisted microwave extraction (VAME) was performed at atmospheric pressure with a microwave frequency of 2450 ± 50 MHz using a microwave extractor (TW/MWEX/2/17/18, Twin Engineers, Pimpri-Chinchwad, India) as shown in Figure 2. This is a multimode microwave reactor with a maximum power output of 1000 W. Microwave absorbing gaskets were used to minimize leakage. The microwave processing cavity dimensions were 900 × 900 × 600 mm (L × W × H). The adapter, condenser, separating funnel, vacuum pump etc. were attached to the extraction setup. 

### 3.3. Extraction of Essential Oil

Solvent-free microwave extraction was performed in a microwave extractor as described by Bayramoglu, Sahin [10]. With the vacuum assisted solvent free microwave extraction (VASFME) process, a 400 g sample of fresh peels of sweet lime was placed in a flat bottom flask, and the apparatus was assembled as shown in Figure 2. The oil extracted from the sample by microwave radiation energy was cooled down in the condenser section. The condensate was channeled out into a separating funnel in order to separate the oil from the water, and a vacuum pump was installed to create suction pressure. In order to examine the process parameters, three different levels of microwave power (500, 750, 1000 W) and extraction time (20, 25, 30 min) were taken. The essential oils were collected, dried over anhydrous sodium sulphate and stored at 4 °C until used [20].

### 3.4. Experiment Design

A central composite design (CCD) was used to investigate the process with a minimal number of possible experiments. This multivariate study allows for the identification of interaction between variables and provides a complete exploration of the studied experiment domain. In this study, yield and antimicrobial activity were taken as responses. The type of CCD used in this study was central composite face centered (CCF). The levels of different process variables are given in Table 1. The application of a CCF design is a convenient way to optimize a three level process (−1, 0, +1) for each factor. The range of different process variables were taken as.

Microwave Power (X_1_): 500–1000 W
Extraction Time (X_2_): 20–30 min

A total of 13 different combinations, including 4 full factorial designs (±1) with 4 axial points (±1) and 5 replicates of the center point (coded 0), were investigated to fit the linear Equation (3)

Y_EO_ = β_0_ + β_1 × 1_ + β_2_X_2_ (3)

where, Y_EO_ represents the response variable (essential oil yield and antimicrobial property in this study), β_0_ is the regression coefficients of variables for intercept and β_1_, and β_2_ are unknown variables. X_1_ and X_2_ are the independent coded variables influencing the response variable Y_EO_. The range of the studied parameters was chosen on the basis of a parametric study, and the results obtained from the CCD were analyzed statistically. The experiment design is shown in Table 1.

### 3.5. Optimization of Extraction Process

In order to investigate the performance of the vacuum assisted solvent free microwave extraction (VASFME) process, an optimization of the operating condition was achieved. The operational parameters viz. microwave power (P) and processing time (t) were chosen for study. Optimization was done by numerical method with the software Design Expert 10 (Version 5.1; Stat-Ease, Inc., Minneapolis, MN, USA) and was carried out for obtaining maximum yield and antimicrobial activity of essential oils extracted from sweet lime peel where the yield and antimicrobial activity of essential oils were maximized and power and time were maintained in range.

### 3.6. Estimation of Yield and Antimicrobial Property of Essential Oil

Essential oils yield was expressed in terms of the volume of the oil collected in milliliters per gram of sweet lime peel (Equation (4)) [10].

(4) $$\mathrm{Yield}\;\left(\%\right)=\;\frac{\mathrm{volume}\;\mathrm{of}\;\mathrm{the}\;\mathrm{oil}\;\mathrm{collected}\;\left(\mathrm{mL}\right)}{\mathrm{weight}\;\mathrm{of}\;\mathrm{sample}\;\mathrm{taken}\;\left(g\right)\;}\;\times 100$$

The antibacterial activity of the essential oils against *Escherichia coli* MTCC 42 (as an indicator microorganism) was determined using an agar well diffusion assay on nutrient agar plates as described by Ahmad and Beg [32]. One vial of 30% glycerol stock of *E. coli* was taken out and streaked in Luria Bertani (LB) agar plate. The plate was kept in 37 °C in an incubator for overnight. One single colony of *E. coli* from the plate was inoculated in 10 mL LB broth and kept in shaking condition at 37 °C overnight. The culture was taken and centrifuged at 4000 rpm for 15 min at 4 °C. The supernatant was removed and the pellet was resuspended with distilled water. The initial optical density of suspension was adjusted to 1 with sterile distilled water. Nutrient agar plates were seeded with 100 μL of overnight grown cultures of target strain and allowed to dry for 10 min. Subsequently, wells of diameter 8 mm were punched into the plates and loaded with 25 μL essential oil. Plates were incubated at 37 °C for 24 h and zones of inhibition were recorded in mm.

### 3.7. Measurement of Physical Properties and Compounds Identification in Essential Oil

Specific gravities and refractive indices of the essential oils extracted by VASFME at optimized condition were measured. Specific gravity was calculated by dividing the weight of 10 µL essential oil by that of 10 µL distilled water. The refractive index was measured 25 ± 2 °C using the Abbe’s refractometer (DR-A1, ATAGO USA Inc., Bellevue, WA 98005, USA).

The essential oils obtained under optimized condition were analyzed by gas chromatography (7890A GC System coupled with 240 Iron trap MS, Agilent Technologies Palo Alto, CA, USA). In order to perform the analysis, capillary column (Agilent 19091J-413 HP5; 30 m × 320 µm × 0.25 µm; Agilent Technologies Palo Alto, CA, USA) was used with a 5% phenyl methyl siloxane stationary phase. GC-MS analysis conditions were as follows: carrier gas: Helium (He), flow rate: 1 mL/min; mode of injection: splitless mode; injection volume: 2 µL; inlet temperature: 250 °C; oven temperature program, from 35 °C to 150 °C with 3 °C/min, holding at 150 °C for 10 min, and then to 250 °C with 10 °C/min; MS transfer line temperature, 250 °C; MS quadrupole temperature, 150 °C; ionization temperature, 230 °C; ionization mode, electronic impact at 70 eV. The solvent delay was 4 min [10]. Identification of compounds was done by comparison with the available National Institute of Standards and Technology (NIST) V2.0 library in the MS system and quantification was done by calculating the percentage area of the obtained peaks in the chromatogram graph.

### 3.8. Ultrasound Assisted Microwave Extraction

The ultrasound pre-treatment was performed in an ultrasonic bath (JSGW, Ambala, India) with a usable capacity of 2.5 L as well as a transducer in the base of the jug, operating at a frequency of 40 kHz and with a maximum input power of 100 W. In a flat bottom flask, sweet lime peels (400 g) with distilled water were placed into an ultrasound bath for 30, 60, 90 and 120 min. Water temperature of bath was maintained at 30 °C by replacing the water at regular interval. After ultrasonic treatment, the flask with sweet lime peels were taken for microwave extraction followed at the optimized condition and the essential oils yield was measured.

### 3.9. Statistical Analysis

Yield and antimicrobial property of essential oil were performed with three replicates, and data were reported as mean ± SD. Duncan’s multiple range tests were used to find significant differences at a significance level of 0.05 using a social sciences SPSS 11.5 (SPSS, Inc., Chicago, IL, USA) statistical package.

## 4. Conclusions

The results demonstrated that the vacuum assisted solvent free microwave extraction method is beneficial for extraction of essential oil from sweet lime peel. Microwave power and extraction time had a significant positive effect on essential oil yield. Whereas, they have shown negative effect on the antimicrobial property of essential oils. Microwave power was shown to exert more effect on both essential oil yield and antimicrobial property compared to extraction time. The optimized values for microwave power and extraction time were 797.844 W and 30 min respectively. The predicted experiment essential oil yield and antimicrobial property were 0.757% and 16.50 mm. In addition, ultrasound pretreatment was found to have a positive impact on essential oil yield. Consequently, the combined effect of ultrasound and microwave extraction may lead to greater essential oil yield. The increase in yield due to ultrasound pretreatment was 37.66%. The extracted essential oil may be useful as an ingredient of food and pharmaceutical industries. The possible future work is to utilize essential oil as a natural antimicrobial agent for application to food or packaging materials.

## Figures and Tables

**Table 1:** Central composite design (CCD) with observed responses for essential oil.

Run No.	Uncoded (Coded) Process Variables		Responses	
	Power (X_1_) W	Time (X_2_) min	Yield (%)	Antimicrobial Property (mm)
1	500 (−1)	20 (−1)	0.60 ± 0.03	33.25 ± 2.94
2	500 (−1)	30 (+1)	0.69 ± 0.07	38.00 ± 3.11
3	750 (0)	25 (0)	0.70 ± 0.06	19.45 ± 1.68
4	750 (0)	20 (−1)	0.63 ± 0.02	23.20 ± 1.91
5	750 (0)	25 (0)	0.70 ± 0.09	25.10 ± 2.06
6	1000 (+1)	30 (+1)	0.79 ± 0.04	4.44 ± 0.81
7	750 (0)	25 (0)	0.70 ± 0.03	24.00 ± 1.99
8	750 (0)	25 (0)	0.71 ± 0.06	23.15 ± 1.97
9	750 (0)	30 (+1)	0.75 ± 0.09	14.60 ± 1.08
10	1000 (+1)	25 (0)	0.77 ± 0.02	7.50 ± 0.96
11	750 (0)	25 (0)	0.69 ± 0.05	19.25 ± 1.74
12	500 (−1)	25 (0)	0.64 ± 0.03	29.05 ± 2.31
13	1000 (+1)	20 (−1)	0.73 ± 0.01	9.64 ± 0.98

**Table 2:** ANOVA for essential oil yield and antimicrobial property.

Parameters		Essential Oil Yield			Antimicrobial Property		
	df	SS	F Value	*p* Value	SS	F Value	*p* Value
Model	2	0.037	94.64	<0.0001	1080.17	40.59	<0.0001
A-Power	1	0.024	125.33	<0.0001	1066.67	80.16	<0.0001
B-Time	1	0.012	63.95	<0.0001	13.50	1.01	0.3376
Residual	10	0.002			133.06		
Lack of Fit	6	0.002	4.17	0.0942	101.06	2.11	0.2458
Pure Error	4	0.0002			32.00		
Cor Total	12	0.039			1213.23		
R-squared		0.95			0.89		
Adj R-squred		0.94			0.87		
CV%		1.99			17.76		

df: degree of freedom; SS: sum of squares; CV: coefficient of variation.

**Table 3:** Effect of ultrasound pre-treatment on essential oils yield.

VASFME		Ultrasound + VASFME		Increase in Yield (%)
Soaking Time (min)	Essential Oil Yield (%)	Ultrasound Treatment Time (min)	EssentialOil Yield (%)	
0	0.77 ± 0.04 ^a^	0	0.77 ± 0.02 ^a^	0
30	0.78 ± 0.02 ^a^	30	0.84 ± 0.03 ^b^	7.69
60	0.78 ± 0.03 ^a^	60	0.91 ± 0.04 ^c^	16.67
90	0.77 ± 0.06 ^a^	90	1.06 ± 0.07 ^d^	37.66
120	0.77 ± 0.05 ^a^	120	0.95 ± 0.06 ^e^	23.38

All data are the mean ± SD of three replicates. Mean followed by different letters (a, b, c, d, e) in the same column differs significantly (*p* ≤ 0.05).

**Table 4:** Composition of the essential oil.

Sl. No.	Compounds	Retention Time (min)	%
1	d-limonene	11.21	43.47
2	bergamol	14.39	7.26
3	β-pinene	9.18	6.84
4	linalool	14.42	5.60
5	α-pinene	7.36	4.49
6	1,8-cineole	11.57	4.05
7	α-terpineol	14.45	3.16
